# Traditional Chinese medicine use is associated with lower end-stage renal disease and mortality rates among patients with diabetic nephropathy: a population-based cohort study

**DOI:** 10.1186/s12906-019-2491-y

**Published:** 2019-04-03

**Authors:** Hsing-Yu Chen, Heng-Chih Pan, Yung-Chang Chen, Yu-Chun Chen, Yi-Hsuan Lin, Sien-Hung Yang, Jiun-Liang Chen, Hau-Tieng Wu

**Affiliations:** 1 Department of Mathematics, University of Toronto, Toronto, Ontario Canada; 2 Graduate Institute of Clinical Medical Sciences, College of Medicine, Chang Gung University, Taoyuan, Taiwan; 3 Division of Chinese Internal Medicine, Center for Traditional Chinese Medicine, Chang Gung Memorial Hospital, Taoyuan, Taiwan; 4 School of Traditional Chinese Medicine, College of Medicine, Chang Gung University, Taoyuan, Taiwan; 5 Division of Nephrology, Department of Internal Medicine, Chang Gung Memorial Hospital, Keelung, Taiwan; 6 Community Medicine Research Center, Chang Gung Memorial Hospital, Keelung, Taiwan; 7 School of Medicine, Faculty of Medicine, National Yang-Ming University, Taipei, Taiwan; 8 Department of Mathematics and Department of Statistical Science, Duke University, 120 Science Dr., 207 Physics Building, Durham, NC 27708 USA; 9 Mathematics Division, National Center for Theoretical Sciences, Taipei, Taiwan

**Keywords:** Chronic kidney disease, Diabetes mellitus, Diabetic nephropathy, End-stage renal disease, Mortality, Traditional Chinese medicine

## Abstract

**Background:**

Diabetic nephropathy (DN) is a common complication of diabetes mellitus (DM) that imposes an enormous burden on the healthcare system. Although some studies show that traditional Chinese medicine (TCM) treatments confer a protective effect on DN, the long-term impact remains unclear. This study aims to examine end-stage renal disease (ESRD) and mortality rates among TCM users with DN.

**Methods:**

A total of 125,490 patients with incident DN patients from 2004 to 2006 were identified from the National Health Insurance Research Database in Taiwan and followed until 2012. The landmark method was applied to avoid immortal time bias, and propensity score matching was used to select 1:1 baseline characteristics-matched cohort. The Kaplan–Meier method and competing-risk analysis were used to assess mortality and ESRD rates separately.

**Results:**

Among all eligible subjects, about 60% of patients were classified as TCM users (65,812 TCM users and 41,482 nonusers). After 1:1 matching, the outcomes of 68,882 patients were analyzed. For the ESRD rate, the 8-year cumulative incidence was 14.5% for TCM users [95% confidence interval (CI): 13.9–15.0] and 16.6% for nonusers (95% CI: 16.0–17.2). For the mortality rate, the 8-year cumulative incidence was 33.8% for TCM users (95% CI: 33.1–34.6) and 49.2% for nonusers (95% CI: 48.5–49.9). After adjusting for confounding covariates, the cause-specific hazard ratio of ESRD was 0.81 (95% CI: 0.78–0.84), and the hazard ratio of mortality for TCM users was 0.48 (95% CI: 0.47–0.50). The cumulative incidence of mortality increased rapidly among TCM users with ESRD (56.8, 95% CI: 54.6–59.1) when compared with TCM users without ESRD (30.1, 95% CI: 29.4–30.9). In addition, TCM users who used TCM longer or initiated TCM treatments after being diagnosed with DN were associated with a lower risk of mortality. These results were consistent across sensitivity tests with different definitions of TCM users and inverse probability weighting of subjects.

**Conclusions:**

The lower ESRD and mortality rates among patients with incident DN correlates with the use of TCM treatments. Further studies about specific TCM modalities or medications for DN are still needed.

**Electronic supplementary material:**

The online version of this article (10.1186/s12906-019-2491-y) contains supplementary material, which is available to authorized users.

## Background

Diabetic nephropathy (DN) is one of the primary causes of end-stage renal disease (ESRD) and accounts for more than 40% of hemodialysis patients [1]. Among all patients with diabetes mellitus (DM), about 25–40% of patients may develop DN 20–25 years after the diagnosis of DM, and about one-third of patients with DN may suffer from ESRD [2]. Because of its high prevalence and severe consequences, DN has been a significant healthcare problem and has resulted in an enormous financial burden [3–5]. In Taiwan, about 6%–8% of adults over 40 years of age have DM, and DN can be found in 40% of adults with DM [6]. This burden may be the primary reason why Taiwan’s healthcare system was ranked 45th out of 195 countries, which is much lower than other developed countries [7]. For this reason, how to provide care that is more comprehensive for patients with DN has become such a vital issue.

Traditional Chinese medicine (TCM) is commonly used by the Chinese population, with prevalence ranging from 45.3% among patients with chronic kidney disease (CKD) [8] to 77.9% among patients with DM in Taiwan [9]. Some TCM treatments may have therapeutic benefits in CKD in clinical studies or DN in animal studies [10–13]. However, studies on ESRD and mortality rates of incident DN in patients with TCM treatments are still lacking since most clinical trials on TCM only reported improvements in renal function. The influence TCM treatments have on ESRD and mortality can only be inferred from the few studies conducted previously in CKD patients but did not differentiate etiology or patients with DM. Nevertheless, the clinical course of incident DN, DM, and CKD may be entirely different in TCM users. Among patients with DM, the risk of DN seemed higher among patients with incident type 2 DM who used TCM [14], but the occurrence of ESRD may be lower among TCM users [15]. However, the mortality rate was not assessed among patients with DM. The mortality rate is not only the most concerned outcome of patients with DN, but the rate of ESRD would also be estimated more accurately when considering mortality [16].

On the other hand, for patients with CKD with undifferentiated etiology, the influence of TCM is somewhat controversial. An early questionnaire-based study revealed that only 10% of patients with CKD ever used TCM, and ESRD risk increased by 20% among TCM users [17]. Some studies showed that certain TCM herbs with aristolochic acid or non-prescribed herbs might be related to renal failure [18–20]. In contrast, a study with data collected from 2000 to 2008, showed that patients with CKD taking aristolochic acid-free TCM herbs had lower risks of mortality [21]. Lin et al. reported that the risk of ESRD was lower among patients with CKD who received TCM herbs during a similar study period without excluding aristolochic acid-containing herbs [8]. However, aristolochic acid-containing TCM treatments may still be a potential confounding factor since only after 2003; all aristolochic acid-containing TCM treatments became strictly prohibited in Taiwan. DN-specific studies aimed to clarify the ESRD and mortality rate of TCM users among patients with DN requires studies to be conducted with a more extensive population and follow-up durations that are free of aristolochic acid-containing herbs [22].

This study aims to explore the mortality and ESRD rates of patients with DN using TCM treatments by studying the entire incident DN cohort from 2004 to 2006, with follow-up until the end of 2012, instead of the sampled dataset used in earlier studies. This information is crucial for both TCM and western medicine (WM) doctors when treating patients with DN.

## Methods

### Data source

The data of this study was obtained from the National Health Insurance Research Database (NHIRD). This database prospectively and routinely stores all medical information of nearly all 23 million inhabitants of Taiwan. Since 1995, the National Health Insurance program (NHI) required both TCM treatments and WM to be entered. Since Taiwan is the only country where the NHI fully reimburses TCM, the NHIRD becomes a unique source to conduct nationwide studies about TCM. All patient information, such as gender, birth date, insured level, residential location, reasons for medical visits, medications, interventions, examinations, hospitalizations, outpatient visits, emergency utilization, and medical expenses, are all digitized and stored in this database. This information provides solutions to important clinical problems about TCM and WM that cannot be resolved easily by conventional designs [23–27].

### Study protocol and subject selections

**Fig. 1:**
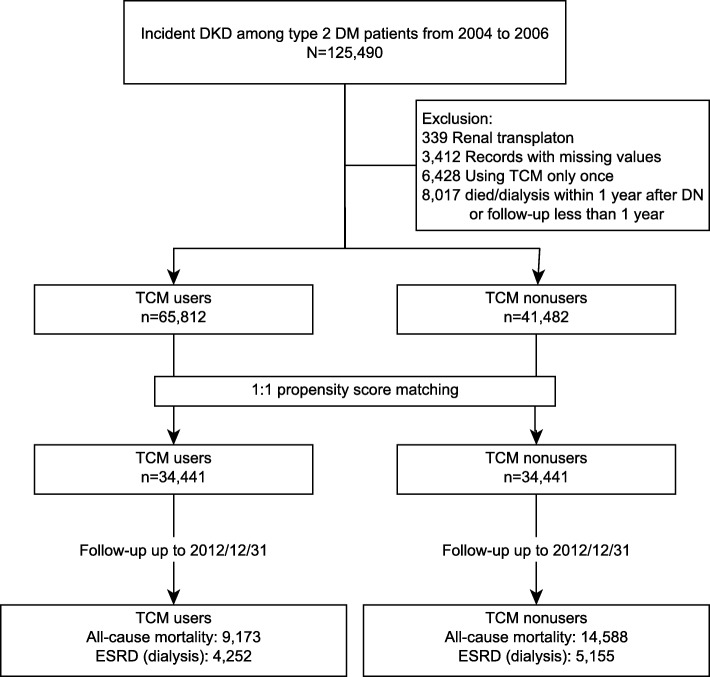
Flow chart of this study. (Abbreviations: DM: diabetes mellitus, DN: diabetic nephropathy, ESRD: End-stage renal disease, TCM: traditional Chinese medicine)

Figure 1 shows the protocol of this study, which was approved by the institutional review board of the Chang Gung Memorial Foundation (No.: 103-1259B). Patients with incident DN between January 1, 2004, and December 31, 2006, were identified. The first day of the DN diagnosis is set as the DN starting point. In Taiwan, the diagnosis of DN was based mainly on the consensus of the Kidney Disease: Improving Global Outcomes (KDIGO) Clinical Practice Guideline [28]. The International Classification of Diseases, 9th Revision, Clinical Modification (ICD-9-CM) was used to recognize the occurrence of DN, and only subjects who were diagnosed at least twice in the outpatient service or once during hospitalization were enrolled (Additional file 1: Table S1) [8, 29]. Subjects with a history of CKD or renal transplantation were excluded.

### TCM users and treatments

We defined TCM users as subjects who received TCM treatments at least twice during the study period, while others were classified as TCM nonusers. Four subgroups were further defined to understand the influence of TCM treatment timing as follows: never TCM users (patients with DN who never used TCM for one year before the DN starting point), former TCM users (received TCM only before the DN starting point), current TCM users (received TCM before and after the DN starting point), and new TCM users (received TCM after the DN starting point). In this case, TCM nonusers included never and former TCM users, while TCM users included current and new TCM users. In addition, to explore the relationships between the duration of TCM use and risks of mortality, we categorized the cumulative duration of TCM use to ≤60, 61–120, 121–180, 181–240, and ≥ 241 days. Patients with only one TCM visit, missing data, previous CKD history, or renal transplantation were excluded.

### Outcomes assessment

Occurrences of ESRD and mortality rates are the outcomes of this study. ESRD occurrence was recognized when the patient received either hemodialysis or permanent peritoneal dialysis. In Taiwan, dialysis is a catastrophic illness, and special payment/management codes are applied (Additional file 1: Table S1). The first date of the occurrence of ESRD was used as the starting date of ESRD. Mortality was recognized when patients permanently withdrew from the insurance program [24, 30]. The day of the outcome occurrence is set as the endpoint. All enrolled patients with DN were followed from DN starting points to the endpoints or the end of 2012.

### Study covariates

Patients’ gender, age, comorbidities, medications, experience with TCM before DN, geolocation, and insured level were used as covariates in this study. Age and insured level were reclassified into categorical variables; four levels for the age factor (less than 20, 21–40, 41–60, and > 60 years), and three levels [0–20,000, 20,001–40,000, and > 40,000 new Taiwan dollar (NTD)/month] for the insured level. The Charlson’s comorbidity index score (CCI) and Diabetes Complications Severity Index (DCSI), with reducing two factors about albuminuria and serum creatine as a modification, were also calculated as summaries of DN-related comorbidities (Additional file 1: Table S1) [31, 32]. Only patients with at least two diagnosis codes in the outpatient service or one during the hospitalization were confirmed as having such comorbidities. Use of diabetic drugs, anti-hypertensive agents, anti-lipid agents, angiotensin converting enzyme inhibitors (ACEi), angiotensin II receptor blockers (ARB), and analgesics was considered when the cumulative duration of treatment was more than 30 days (Additional file 1: Table S2). The use of medications for comorbidities was used as the proxy for quality control of the comorbidities of each patient [33].

### Bias assessment

The use of the whole population cohort extracted from a population-based database that was prospectively collected can substantially alleviate possible recall bias, selection bias, and referral bias when compared with studies using a hospital-based, sample database or questionnaires [34, 35]. The landmark method was conducted to avoid immortal time bias by excluding the subjects who died or entered the ESRD stage within one year after diagnosis since the mean interval between the DN diagnosis and initiation of TCM treatment was about 240 days in this cohort. Detection bias might become a problem when DN was mainly diagnosed by measuring renal function and albuminuria in the clinical setting [36]. Since there were no lab data recorded in the NHIRD, we validated the DN patient recognition procedure by examining an external hospital-based database, acquired from the Chang Gung Memorial Hospital, Linkou, Taiwan. The positive predictive value of the same protocol for the DN patient recognition procedure was 97.5% in the hospital-based database, which indicated that the risk of detection bias was low and acceptable (Additional file 1: Table S3).

### Statistical analysis

Descriptive statistics were used to demonstrate the baseline characteristics of patients with DN. Categorical data are presented as numbers or counts with percentage, and continuous data, such as the duration of TCM use and the follow-up duration, are presented as means with standard deviations or with 95% confidence intervals (CI). Because the initial status of patients with incident DN may be a confounding factor to the outcome, propensity score matching (PSM) was applied to eliminate baseline differences between TCM users and nonusers when DN was diagnosed, including genders, ages, medications for DM and hypertension, insured level, geolocation, previous TCM use experience, and the severity of DM-related complications. All these viable covariates were used in the multiple logistic regression model to generate a score for each patient, and each eligible subject was matched according to the score by using the nearest-neighbor method at a 1:1 ratio with 0.2 in caliper and without subject replacement as suggested [37]. Another matched cohort was acquired of a smaller caliper to minimize risks of selection and confounding bias. Both cumulative incidence and incidence rate (IR) were reported for both ESRD and mortality rates. For estimating the cumulative incidence of mortality, the Kaplan–Meier method was applied, and the competing-risk analysis was performed to study the cumulative incidence of ESRD. The competing-risk analysis may estimate the ESRD rate more accurately when considering the pre-ESRD mortality as the competing event against ESRD [16]. The hazard ratio (HR) was calculated under the Cox regression model for mortality, and the cause-specific hazard ratio (CSHR) was estimated by the competing-risk regression for ESRD occurrence. All covariates mentioned above, including medication use and comorbidities, were used in both regression models to adjust for possible influences of covariates on outcomes.

A subgroup analysis was also conducted, including age groups (reclassified as < 60 and ≥ 60 years), gender, duration and status of TCM use, and DN-related comorbidities. The DCSI score was reclassified as < 2 and ≥ 2 points, whereas the CCI score was categorized as < 4 and ≥ 4. Moreover, the mortality rate was estimated among patients with and without the occurrence of ESRD. Finally, we performed sensitivity tests using different study cohorts and sample weights. Study cohorts were redefined as follows: (1) the whole population without excluding patients who died or had ESRD within one year after DN or used TCM only once, (2) TCM users without patients initiating TCM treatment within 6 months before death or end of follow-up, and (3) redefined TCM users by cumulative TCM use duration ≥30, 60, and 90 days in addition to using TCM at least twice. Also, the inverse probability weight for the entire population was used to estimate the risks of mortality [38]. All statistical analyses were done using the software STATA (version 14.0), in which the package stcox was used for Cox regression, and compet/stcrreg were used for competing-risk analysis. Statistical results with *p*-values <.05 were considered significant. The Bonferroni’s correction was applied if multiple comparisons were conducted.

## Results

### Demographic characteristics of diabetic nephropathy patients

A total of 107,294 patients with incident DN were identified, of whom 66,851 were TCM users, and 43,428 were nonusers (Fig. 1). Although more than 60% of patients received TCM treatments after diagnosis, only 2477 TCM users (3.7% of all users) received TCM treatments alone. The average time from the DN starting point to the initiation of TCM treatment was 240.2 days with an average duration of 1526 days. The baseline demographic characteristics of TCM users were quite different from nonusers (Additional file 1: Table S4). The differences between TCM users and nonusers were adequately resolved using PSM, and the matched cohort with 68,882 patients was further analyzed (Table 1).

**Table 1:** Comparable demographic features among TCM users and non-TCM users after 1:1 propensity score matching

	TCM users (*n* = 34,441)		TCM nonusers (*n* = 34,441)		Standardized mean difference
Gender					0.068
Female	14,455	(42.0%)	15,623	(45.4%)	
Male	19,986	(58.0%)	18,818	(54.6%)	
Age (years)					−0.017
-20	24	(0.1%)	49	(0.1%)	
21–40	716	(2.1%)	1072	(3.1%)	
41–60	8429	(24.5%)	10,140	(29.4%)	
61-	25,272	(73.4%)	23,180	(67.3%)	
Insured level (NTD/month)					−0.068
0–20,000	30,012	(87.1%)	28,832	(83.7%)	
20,001–40,000	2330	(6.8%)	3343	(9.7%)	
40,001–	2099	(6.1%)	2266	(6.6%)	
Geolocation					0.030
1 (more urban)	8495	(24.7%)	8648	(25.1%)	
2	9400	(27.3%)	9630	(28.0%)	
3	5173	(15.0%)	5311	(15.4%)	
4	6273	(18.2%)	6058	(17.6%)	
5	1063	(3.1%)	1020	(3.0%)	
6	2157	(6.3%)	2028	(5.9%)	
7 (more rural)	1880	(5.5%)	1746	(5.1%)	
Previous TCM users	868	(2.5%)	2962	(8.6%)	−0.181
Comorbidities					
Hypertension	22,578	(65.6%)	21,575	(62.6%)	0.060
Hyperlipidemia	10,659	(30.9%)	10,979	(31.9%)	−0.020
Heart failure	2147	(6.2%)	2030	(5.9%)	0.015
IHD	7123	(20.7%)	6867	(19.9%)	0.019
CVD	3143	(9.1%)	3016	(8.8%)	0.013
Hyperuricemia	4242	(12.3%)	4060	(11.8%)	0.017
COPD	4207	(12.2%)	4180	(12.1%)	0.002
CCI	4.2	(1.9)	4.0	(2.0)	0.088
Modified DCSI score	1.5	(1.3)	1.4	(1.3)	0.035
Confounding drugs					
Diabetic drugs					
Insulin analogs	3716	(10.8%)	3534	(10.3%)	0.018
Biguanides	19,275	(56.0%)	19,019	(55.2%)	0.015
SU	22,281	(64.7%)	21,990	(63.8%)	0.018
Alpha-glucosidase inhibitors	3597	(10.4%)	3577	(10.4%)	0.002
TZD	4560	(13.2%)	4597	(13.3%)	−0.003
Others	2612	(7.6%)	2523	(7.3%)	0.010
Lipid-lowering agent					
Statin	8342	(23.3%)	8291	(23.2%)	0.005
Fibrate	4003	(11.2%)	4000	(11.2%)	0.000
Others	104	(0.3%)	90	(0.3%)	0.008
Anti-hypertensives					
ACEi	9369	(27.2%)	8976	(26.1%)	0.026
ARB	9511	(27.6%)	9029	(26.2%)	0.032
α-blocker	2653	(7.7%)	2452	(7.1%)	0.023
β-blocker	10,484	(30.4%)	10,181	(29.6%)	0.019
CCB	16,008	(46.5%)	15,137	(44.0%)	0.051
Diuretics	10,402	(30.2%)	9816	(28.5%)	0.038
Vasodilator	4640	(13.5%)	4464	(13.0%)	0.015
Central-acting agent	2653	(7.7%)	2452	(7.1%)	0.027
Analgesics					
NSAID	10,429	(30.3%)	10,839	(31.5%)	−0.025
COX-2 inhibitors	1785	(5.2%)	1685	(4.9%)	0.014
Acetaminophen	8672	(25.2%)	9103	(26.4%)	−0.028
Aspirin	11,949	(34.7%)	11,574	(33.6%)	0.023

Abbreviations: *ACEi* angiotensin converting enzyme inhibitor, *ARB* angiotensin II receptor blocker, *CCB* calcium channel blocker, *CCI* Charlson’s comorbidity index, *COPD* chronic obstructive pulmonary disease, *COX-2* cyclooxygenase-2 inhibitor, *DCSI* Diabetes Complications Severity Index, *NSAID* nonsteroidal anti-inflammatory drug, *NTD* new Taiwan dollar, *SU* Sulfonylureas, *TCM* traditional Chinese medicine, *TZD* Thiazolidinediones

### TCM users associated with lower ESRD and mortality rates

**Fig. 2:**
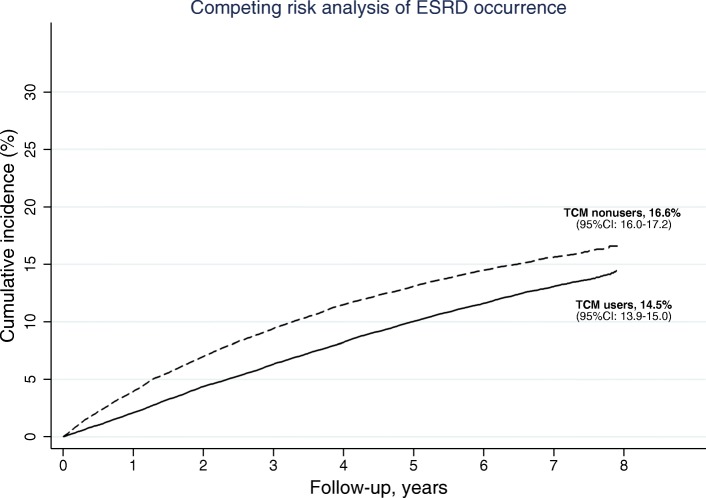
Competing-risk analysis of the ESRD rate in the matched cohort, by TCM users and nonusers

The ESRD rates were lower among TCM users compared with nonusers. Before matching, the overall IR of ESRD was 20.2 per 1000 patient-years (PY), and 22.71000 PY after matching (Table 2). The overall 8-year cumulative incidence of ESRD was 14.4% (95% CI: 14.1–14.7). The IR of ESRD among TCM users was lower than that of TCM nonusers (19.2 versus 26.7 per 1000 PY for TCM users and TCM nonusers after matching, respectively). Also, the cumulative incidence was lower among TCM users (14.5, 95% CI: 13.9–15.0) than nonusers (16.6, 95% CI: 16.0–17.2, Fig. 2). When considering all potential confounding covariates in the competing regression model, the risk of ESRD among TCM users was reduced by 19% (aCSHR: 0.81, 95% CI: 0.78–0.84, *p*-value <.001).

**Table 2:** Incidence rates and risks of ESRD and mortality among TCM users and TCM nonusers

	Overall			TCM user			TCM nonuser			
	Case	PY	I^a^	Case	PY	I^a^	Case	PY	I^a^	aHR/aCSHR^b^ (95% CI)
Before matching										
All-cause mortality	32,201	703,192.6	47.4	14,048	458,457	30.6	18,153	244,735.7	74.1	0.48 (0.47–0.49)*
ESRD	13,538	669,671.5	20.2	7363	439,654.1	16.7	6175	230,017.3	26.8	0.74 (0.72–0.77)*
After matching										
All-cause mortality	23,761	437,362.7	54.3	9173	232,106.2	39.5	14,588	205,256.5	71.1	0.48 (0.47–0.50)*
ESRD	9407	414,628.6	22.7	4252	221,755.2	19.2	5155	192,873.3	26.7	0.81 (0.78–0.84)*

Abbreviations: *aCSHR* adjusted cause-specific hazard ratio, *aHR* adjusted hazard ratio, *ESRD* end-stage renal disease, *TCM* traditional Chinese medicine;

*p-value <.001

^a^Incidence is presented as 1000 person-year (PY)

^b^Age, gender, geolocation, insurance level, comorbidities, medications, and previous TCM experience, were adjusted in the Cox regression model to evaluate the adjusted hazard ratio (aHR) for all-cause mortality. Age, gender, geolocation, insured level, comorbidities, medications, previous experience with TCM were fitted in the competing-risk regression to evaluate the adjusted cause-specific hazard ratio (aCSHR) for ESRD

**Fig. 3:**
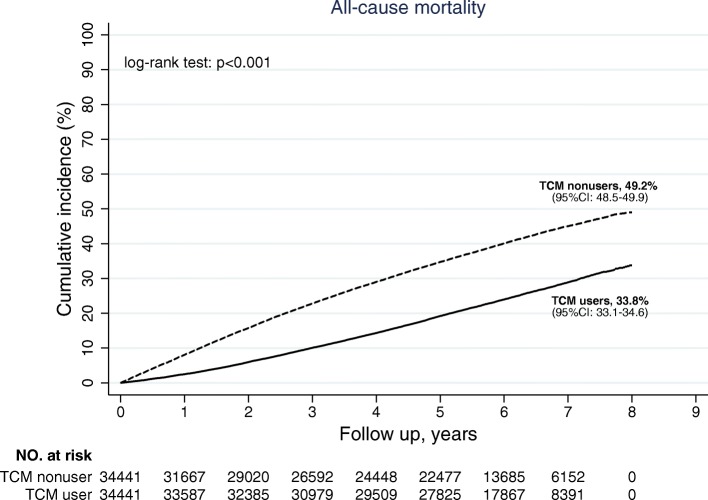
Survival analysis of mortality rate in the matched cohort, by TCM users and nonusers

In addition to the lower ESRD rate, the mortality rate was lower among TCM users to a larger degree. Before matching, the overall IR of mortality was 47.4 per 1000 PY, and the 8-year cumulative incidence was 36.0% (95% CI: 35.6–36.4). The IR of mortality was 39.5 per 1000 PY for TCM users and 71.1 per 1000 PY for TCM nonusers (Table 2). The cumulative incidence of mortality among TCM users was 33.8% (95% CI: 33.1–34.6) and about 16% lower than TCM nonusers (59.2, 95% CI: 48.5–49.9, log-rank test *p*-value <.001, Fig. 3). After adjusting for potential confounding factors, the mortality risk was reduced by 52% among TCM users (aHR: 0.48, 95% CI: 0.47–0.50, *p*-value <.001).

### The association of duration and initiation times of TCM use with mortality

We found lower mortality risks among TCM users are duration-dependent, that the longer duration of TCM use was associated with both lower mortality rate and aHR (Table 3). In addition, the starting time of TCM treatment had a significant influence on mortality risk. Among all TCM users, nearly 60% of patients continued TCM treatment after the DN starting point (current TCM users), and others initiated TCM treatment after the DN starting point (new TCM users). Both current and new TCM users had lower mortality rates compared with never TCM users (aHR: 0.47, 95% CI: 0.46–0.49 for new TCM users, and aHR: 0.49, 95% CI: 0.48–0.51 for current TCM users, both log-rank tests *p* < .001). Patients who used TCM treatments only before DN (former TCM users) shared a similar risk of mortality with never TCM users.

**Table 3:** Mortality associated with the TCM users, by duration of TCM use and TCM use status

	Death	PY	I^a^	Before matched		After matched	
				aHR^$^ (95% CI)	Sig.	aHR^$^ (95% CI)	Sig.
Duration of TCM use (days)							
TCM nonuser	14,588	170,815.5	85.4	1 (reference)		1 (reference)	
≤ 60	6144	114,886.8	53.5	0.55 (0.54–0.57)	***	0.56 (0.54–0.57)	***
61–120	1236	27,672.5	44.7	0.47 (0.44–0.49)	***	0.47 (0.44–0.50)	***
121–180	566	13,663.0	41.4	0.40 (0.37–0.43)	***	0.43 (0.39–0.46)	***
181–240	311	8969.5	34.7	0.36 (0.33–0.40)	***	0.36 (0.32–0.40)	***
241–	916	32,473.4	28.2	0.29 (0.27–0.30)	***	0.29 (0.28–0.31)	***
TCM use status							
Never user	10,699	127,149.0	84.1	1 (reference)		1 (reference)	
Former user	3889	43,666.6	89.1	1.01 (0.98–1.05)		1.02 (0.98–1.06)	
Current user	5530	113,624.6	48.7	0.49 (0.47–0.50)	***	0.49 (0.48–0.51)	***
New user	3643	84,040.59	43.3	0.47 (0.45–0.48)	***	0.47 (0.46–0.49)	***

*Significance: *p-value <.05; **p-value <.01; ***p-value <.001

^a^Incidence is presented as 1000 person-year (PY)

^$^Age, gender, geolocation, insured level, comorbidities, medications, previous TCM experience, were adjusted in the Cox regression model to evaluate the hazard ratio for all-cause mortality

### Subgroup analysis on the risk of mortality

**Fig. 4:**
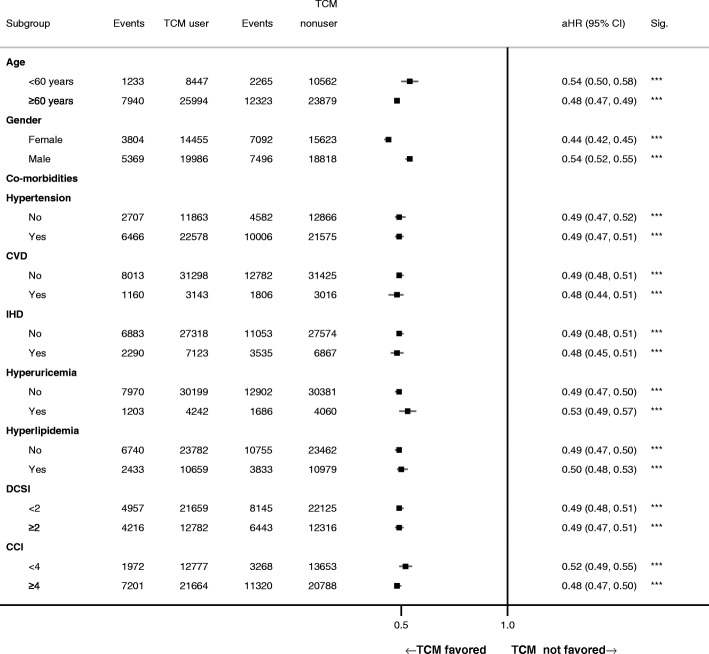
Multivariate subgroup analysis for the impact of TCM use on all-cause mortality. Abbreviations as in Table 1. *Significance: *p-value <.05; **p-value <.01; ***p-value <.001. ^$^Cox regression model with adjusted covariates, including age, gender, geolocation, insured level, comorbidities, medications, and previous TCM experience. Each covariate listed above was excluded from the subgroup analysis itself but included in the subgroup analysis with other covariates

**Fig. 5:**
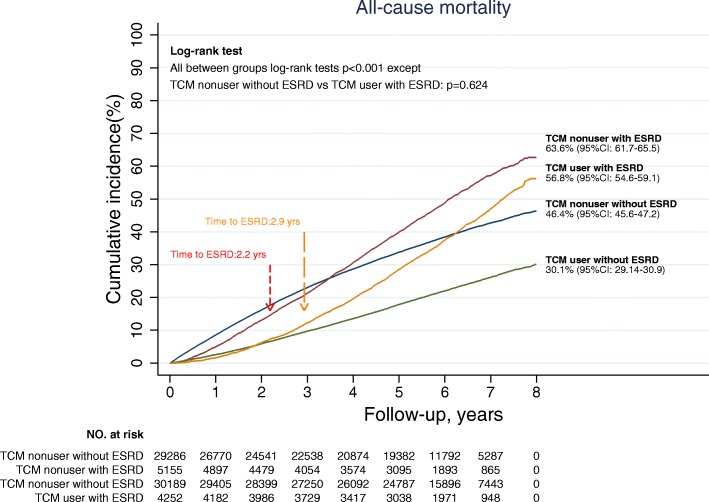
Cumulative incidence of mortality stratified by TCM use and ESRD occurrence

The subgroup analysis showed that TCM users had lower risks of mortality than those of nonusers among stratifications of age, gender, and comorbidities (Fig. 4). Moreover, among patients with ESRD, the mortality rate was higher than patients without ESRD (Fig. 5). Although the mortality rate of TCM users was still lower than that of nonusers among patients with ESRD (8-year cumulative incidence reduced 6.8% among TCM users, log-rank test *p*-value <.001), the reduction in mortality rate was much lower than in patients without ESRD (8-year cumulative incidence reduced 16.3% among TCM users, log-rank test *p*-value <.001). After adjusting for covariates, the aHR of mortality among TCM users was higher with the occurrence of ESRD (aHR: 0.73, 95% CI: 0.68–0.77) than without ESRD (aHR: 0.50, 95% CI: 0.48–0.51).

### Sensitivity tests

The sensitivity tests firmly supported the results. Among the full cohort, the same study cohort with inverse probability-weighted subjects, or the cohort with a different definition of TCM user selected from patients with incident DN, we concluded that TCM users had a lower mortality risk compared with TCM nonusers (Table 4). Moreover, the results were similar in the two cohorts matched by PSM with different calipers. The differences between TCM users and nonusers were lower in the matched cohort with a low caliper (Additional file 1: Table S5, Figures S1, and S2).

**Table 4:** Sensitivity analyses on risks of mortality among TCM users^a^

Model	aHR (95% CI)^a^	Sig.
Full cohort (*n* = 121,739)^b^	0.49 (0.48–0.50)	***
Same study cohort with inverse probability weighting (*n* = 107,294)	0.51 (0.49–0.52)	***
Redefine TCM users^c^		
Excluding late TCM users (*n* = 67,604)	0.48 (0.47–0.49)	***
Excluding former TCM users		
Cumulative duration ≥30 days (*n* = 51,568)	0.40 (0.39–0.42)	***
Cumulative duration ≥60 days (*n* = 41,974)	0.37 (0.36–0.39)	***
Cumulative duration ≥90 days (*n* = 35,252)	0.35 (0.33–0.36)	***

*Significance: *p-value <.05; **p-value <.01; ***p-value <.001

^a^The same covariates, including age, gender, geolocation, insured level, comorbidities, medications, and previous experience with TCM were adjusted in every model

^b^only excluding renal transplantation patients and missing values

^c^TCM users were redefined as followings: all TCM users except patients who initiated TCM treatment 6 months before death/end of follow-up (late TCM users), or patients who used TCM longer than 30, 60, or 90 days. PSM was used to select baseline characteristics-matched cohorts

## Discussion

This study is the first and most extensive cohort study about mortality and ESRD rates of TCM users who are patients with incident DN. Although observational studies are not as rigorous as randomized clinical trials for assessing effectiveness and causality, this study shows that lower ESRD and mortality rates highly correlated with the use of TCM. Both of these long-term outcomes are consistent with the short-term outcomes mentioned in previous clinical and cohort studies [12, 15, 39]. The use of a cohort from 2004 enabled us to both remove any potential adverse effects of aristolochic acid-containing herbs and estimate the outcomes of patients with DN who used TCM more accurately than previous studies [8, 21]. The TCM treatment is unlikely the primary factor contributing to patients with DN who were unsatisfied with their healthcare mentioned recently [7], nor the precipitating factor reported previously among patients with all-cause CKD [17–19, 40]. Instead, reduced ESRD and mortality rates among TCM users showed the potential of TCM treatments to be considered as a part of the integrative care system for patients with DN. Also, the results provide crucial information about using TCM for DN, since previous clinical studies still lack data regarding changes in ESRD or mortality rates because of the limited duration of follow-ups [12, 39].

The potential renoprotective effect may be the reason why the all-cause mortality rate was lower among TCM users since the ESRD rate was also lower and the time to ESRD was about one year later than among TCM nonusers. These results closely correspond to several clinical trials that examined the efficacy of TCM treatments on DN, in which proteinuria and the glomerular filtration rate improved by integrating TCM treatments into standard WM treatments [39]. Renoprotection may come from direct effects, such as *Astragalus membranaceus* (Fisch.) Bge.*,* Huang Qi in Chinese, for improving proteinuria [12], or decreasing the use of nephrotoxic WM medications [41, 42].

Nevertheless, renoprotection may be only one reason that TCM users had a lower mortality rate since the risks of mortality decreased much more than the risks of ESRD. Because DN may solely increase the risks of cardiovascular diseases [43], cerebrovascular diseases [44], and even various kinds of cancer [45], both the clinical courses and treatment effects among patients with DN with these complications may be different [43]. For this reason, the potential of TCM treatments for reducing the risk of these complications and mortality rates among patients with DN is worthy of further study, especially when TCM users were associated with better outcomes among patients with stroke or malignancies [46, 47]. Furthermore, we also found that the occurrence of ESRD might compromise the lower mortality rate among TCM users. Patients with ESRD or even pre-ESRD were thought to have less residual renal function, which may cause a higher mortality rate than patients without ESRD. Since studies about TCM use among patients with ESRD are limited and only certain TCM treatments seemed beneficial among patients with ESRD [48, 49], further studies about long-term outcomes of TCM users among patients with pre-ESRD or ESRD patients are needed.

In addition, the influence of the time of initiating TCM treatments implies that TCM doctors need to consider DN earlier, even before it occurs. TCM users who started the TCM treatment only after DN diagnosis, namely new TCM users, had the best survival rate, while the mortality of former TCM users was the highest among all patients with DN. The different outcomes of patients with various initiation times suggest that TCM doctors may consider protecting renal function even before the diagnosis of DN as the currently recommended management for DN [50]. The lower risks of mortality among new TCM users implies that some TCM treatments might have secondary protective effects as a part of the integrative management of patients with DN since some TCM treatments may improve renal function and proteinuria when combined with conventional WM treatments [12, 51]. On the contrary, some TCM treatments should be used cautiously in patients with DM even before DN occurs, as former TCM users showed in our study. For example, TCM treatments intended to remove excess body fluid may cause fluid imbalance or even dehydration, which may potentially damage renal function [8].

Moreover, as the initial report about the risks of ESRD and mortality among patients with incident DN in the Chinese population (the main ethnic group in Taiwan), we found that the results were entirely different from risks assessed among patients with CKD of undifferentiated etiology. The ESRD rate among DN was twice as high as in patients with general CKD in Taiwan (IR: 20.2 per 1000 PY for DN versus 11.1 per 1000 PY for all CKD re-calculated according to Lin et al.’s report [8]). For TCM users, the risk of ESRD reduced less among patients with DN (aCSHR: 0.81 for DN versus 0.47 for all CKD [8]), but the risk of mortality seemed comparable (aHR: 0.48 versus 0.6 for all CKD [21]). Since CKD may have various causes, the influence of TCM treatments would vary widely depending on the causes, and therefore, the outcomes of TCM users should be estimated accordingly.

Through this study, we demonstrated that TCM users had lower ESRD and mortality rates among patients with incident DN. For this reason, the use of TCM should be assessed when visiting patients with DM, and it also should be taken into consideration when conducting cohort studies about DN. However, there are some limitations to this study. First, since only reimbursed TCM treatments were included in this study, we may have underestimated the use of TCM because of the lack of data regarding self-paid TCM and folk medicines. Since TCM treatments are reimbursed and readily accessible (the medication fee is about 1 USD/per day), the influence caused by the self-paid TCM and folk medicine would be minimal. Besides, the efficacy of specific TCM treatment was not assessed in this study since it is unlikely to use all kinds of TCM treatments as a therapeutic regimen for DN. In this study, we included all kinds of TCM treatments under the consideration that the guideline or consensus of TCM treatments for DN are still lacking, and TCM prescriptions are somewhat complicated in the real world due to the TCM treatment theory “bian-zheng-lun-zhi,” which means that treatments should be personalized according to the individuals’ conditions. Therefore, the feasibility of using TCM treatments among patients with DN assessed in this study would be helpful when intending to explore the effectiveness of specific TCM treatments by conducting clinical trials or bench studies. Second, although PSM was used to decrease the differences between TCM users and nonusers, it is impossible to assess all potential confounding factors for ESRD or mortality. For example, TCM users are usually associated with higher socioeconomic status (as the unmatched cohort in our study), and therefore the lower risk may be relevant to both TCM use and socioeconomic status. In addition to choosing a matched cohort by using the geolocation and insured level as the proxy for socioeconomic status [8, 25], we also found former TCM users had an inconsistent outcome with other TCM users (Table 3), which may imply that bias from socioeconomic status may be minimal. Third, since the information about stopping treatment is not available in this database, we do not know the exact reason why former TCM users discontinued TCM treatment before DN diagnosis. One possible reason is deteriorating renal function, and discontinuation of treatment may be suggested at that time. Because the average treatment effect of TCM seems beneficial in this study, and the causative nephrotoxic agents have been prohibited since 2003, TCM treatments are unlikely the leading cause of poor outcomes. Instead, late diagnosis of DN may be one crucial possible factor since the overall outcome of patients with incident DN seemed poorer than western countries. Another concern is that the poorer outcomes of former TCM users may indicate that nephrologists may stop some TCM treatments because of deteriorating renal function. However, this reason for discontinuation could not be verified because it was not accessible, and the lab data was not available in this database. Theoretically, this condition may confound the outcome analysis, but since the HR was only 0.01 higher than patients who never used TCM and the HR was much lower among new TCM users, we proposed positive correlations between TCM use and a better survival rate.

Nevertheless, we still suggest that TCM doctors should be cautious about patients’ renal function and choose TCM treatments carefully even before a definite diagnosis of DN is made. Fourth, the actual quality of control in DM and hypertension is crucial to patients with DN, but the relevant laboratory data is absent in this database. Extensive consideration of potential confounding medications (e.g., medications for hypertension and DM), the severity of DN-related complications (e.g., DCSI and CCI), the CKD-related complications (e.g., hyperuricemia, and cardiovascular disease), and renoprotective agents, may enable this limitation to be overcome for the most part. Finally, some newly approved anti-diabetes medications are not included in this study, such as the sodium-glucose cotransporter-2 inhibitors. This medication can lower cardiovascular risk among patients with DM and may decrease the mortality rate. However, it was not possible to include this medicine in the analysis since it was only approved in Taiwan in 2014.

## Conclusions

We demonstrated that TCM users might have a lower mortality and ESRD rate among patients with incident DN in the Chinese population. Previously published clinical trials reported that various TCM modalities and medications have short-term renoprotective effects (lowering proteinuria and renal dysfunction). This study provides additional results that suggest possible long-term outcomes of TCM users (the occurrence of death and ESRD). However, since then, all patients with any TCM treatments were included as TCM users, and the observational cohort study was not the gold standard for assessing the effectiveness of specific treatments. Further studies, including well-designed, clinical trials and pharmacology studies about the renoprotective effects of specific TCM modalities and medications, are still needed.

## Additional file

Additional file 1: **Table S1.** Diagnosis codes used in the study. **Table S2.** Medications codes used in this study. **Table S3.** The CKD stage and albuminuria of DN patients when diagnosed in the Department of Nephrology, Chang Gung Memorial Hospital, Taiwan, from 2004 to 2012. (*n* = 5384 with 136 normoalbuminuria patients). **Table S4.** Demographic and medical history of patients with incident diabetic nephropathy during 2004–2006 (*N* = 107,294). **Table S5.** Comparable demographic features among TCM users and non-TCM users after 1:1 propensity score matching, in which caliper was set to 0.00001. The standard mean differences between two groups become smaller than the cohort matched by PSM with caliper 0.2 under this condition. **Figure S1.** Competing analysis on ESRD in the matched groups, by TCM users and nonusers. **Figure S2.** Survival analysis on mortality rate in the matched groups, by TCM users and nonusers. (DOCX 335 kb)

## Abbreviations

ACEi: Angiotensin converting enzyme inhibitor
ARB: Angiotensin II receptor blocker
CCB: Calcium channel blocker
CCI: Charlson’s comorbidity index
CI: Confidence interval
CKD: Chronic kidney disease
COPD: Chronic obstructive pulmonary disease
COX-2: Cyclooxygenase-2
CSHR: Cause-specific hazard ratio
DCSI: The Diabetes Complications Severity Index
DM: Diabetes mellitus
DN: Diabetic nephropathy
DPP4i: Dipeptidyl peptidase-4 inhibitor
EPO: Erythropoietin
ESRD: End-stage renal disease
HR: Hazard ratio
ICD-9-CM: The International Classification of Diseases, 9th Revision, Clinical Modification
IR: Incidence rate
NHI: The National Health Insurance
NHIRD: The National Health Insurance Research Database
NSAID: Nonsteroidal anti-inflammatory drug
NTD: New Taiwan dollar
PSM: Propensity score matching
PY: Person-years
SU: Sulfonylurea
TCM: Traditional Chinese medicine
TZD: Thiazolidinediones
WM: Western medicine
