# Supplementary material for: Traditional Chinese medicine use is associated with lower end-stage renal disease and mortality rates among patients with diabetic nephropathy: a population-based cohort study
Source: BMC Complement Altern Med. 2019 Apr 3;19:81. doi: 10.1186/s12906-019-2491-y (PMC6448220; doi:10.1186/s12906-019-2491-y)

**Table S1.** Diagnosis codes used in the study

| Disease Name | ICD-9-CM codes |
| --- | --- |
| Type 2 diabetes mellitus | 250.x, except 250.x1 and 250.x3 |
| Chronic kidney disease | 580.x-588.x, 250.4x, 274.1x, 283.11, 403.x1, 404.x2, 404.x3, 440.1, 442.1, 447.3, 572.4, 642.1x, 646.2x and 791.0 |
| End-stage renal disease (permanent dialysis) | 39.95, v45.1, v56.0, and v56.8, catastrophic illness code “03” |
| Ischemic heart disease | 410, 411, 412 |
| Heart failure | 401.91, 402.01, 402.11, 404.01, 404.03, 404.11, 404.91, 404.93, and 428 |
| Hypertension | 401-405 |
| Hyperlipidemia | 272 |
| Hyperuricemia | 274 |
| Cerebrovascular disease | 430-438 |
| Chronic Obstructive Pulmonary Disease | 491, 492, and 496 |

Abbreviation: ICD-9-CM, International Classification of Disease, 9^th^ Revision, Clinical Modification

**Table S2.** Medications codes used in this study

| Medicine Name | ATC codes |
| --- | --- |
| Angiotensin converting enzyme inhibitor (ACEi) | C09C, C09D |
| Angiotensin II Receptor Blockers (ARB) | C09A, C09B |
| Direct renin inhibitors (DRI) | C09XA |
| α-blocker | C02CA |
| β-blockers | C07 |
| Calcium channel blockers (CCB) | C08C, C08D |
| Central-acting agents | C02 |
| Vasodilators | C04 |
| Diuretics | C03 |
| Insulin | A10A |
| α-glucosidase inhibitor | A10BF |
| Biguanides | A10BA |
| Sulfonylureas (SU) | A10BB |
| Dipeptidyl peptidase-4 inhibitor (DPP4i) | A10BH |
| Glucagon-like peptide-1 | A10BX07, A10BX14, A10BJ02, A10BX04, A10BJ01 |
| Thiazolidinedione (TZD) | A10BG |
| Other hypoglycemic agents | A10BD, A10BX |
| Statin | C10AA, C10B |
| Fibrate | C10AB |
| Other lowering lipid agents | C10AC, C10AD, C10AX |
| Acetaminophen | R05X, N02BE51, M03BC51, N02BE01, M03BB53, M03BB52, N02BE71, N02BA57 |
| Nonsteroidal anti-inflammatory drug (NSAID) | M01AA, M01AB, M01AC, M01AE, M01AG, M01AX |
| Selective COX-2 inhibitors | M01AH |
| Aspirin | J05AX, N02BA01, B01AC06, M01BA03, N02BA51, B01AC04, B01AC30, R05X |
| Erythropoietin (EPO) | B03XA01, B03XA02, B03XA03 |

**Table S3.** The CKD stage and albuminuria of DN patients when diagnosed in the Department of Nephrology, Chang Gung Memorial Hospital, Taiwan, from 2004 to 2012. (n = 5384 with 136 normoalbuminuria patients)

Description of data: Validation of the study protocol to identify patients with DN by applying the same protocol to the hospital-based database.

|  |  |  | Albuminuria (mg/g)^$^ | |
| --- | --- | --- | --- | --- |
| CKD stage* | Subjects | Percentage | mean | ±SD |
| 1 | 31 | 0.58 | 1,442.72 | ±4,123.09 |
| 2 | 649 | 12.05 | 665.91 | ±1,436.48 |
| 3a | 676 | 12.56 | 1,141.13 | ±2,176.83 |
| 3b | 1,151 | 21.38 | 1,414.36 | ±2,265.08 |
| 4 | 1,505 | 27.95 | 2,963.59 | ±5,802.20 |
| 5 | 1,236 | 22.96 | 3,746.97 | ±2,724.54 |
| *CKD stage was classified by estimated glomerular filtration rate calculated according to the modification of diet in renal disease equation  ^$^Albuminuria was calculated by urine albuminuria-creatinine ratio, < 30 mg/g regarded as normoalbuminuria | | | | |

**Table S4**. Demographic and medical history of patients with incident diabetic nephropathy during 2004–2006 (N = 107,294)

|  | | | TCM users  (n= 65,812) | | TCM nonusers  (n= 41,482) | | *p*-value |
| --- | --- | --- | --- | --- | --- | --- | --- |
| Gender | | |  |  |  |  | <0.001 |
|  | | Female | 33,524 | (50.9%) | 17,985 | (43.4%) |  |
|  | | Male | 32,288 | (49.1%) | 23,497 | (56.6%) |  |
| Age (years) | | |  |  |  |  | <0.001 |
|  | | -20 | 151 | (0.2%) | 56 | (0.1%) |  |
|  | | 21-40 | 3,192 | (4.9%) | 1,189 | (2.9%) |  |
|  | | 41-60 | 26,298 | (40.0%) | 11,502 | (27.7%) |  |
|  | | 61- | 36,171 | (55.0%) | 28,735 | (69.3%) |  |
| Insured level (NTD/month) | | |  |  |  |  | <0.001 |
|  | | 0-20,000 | 50,681 | (77.0%) | 35,014 | (84.4%) |  |
|  | | 20,001-40,000 | 9,613 | (14.6%) | 3,822 | (9.2%) |  |
|  | | 40,001- | 5,518 | (8.4%) | 2,646 | (6.4%) |  |
| Geolocation | | |  |  |  |  | <0.001 |
|  | | 1 (more urban) | 15,317 | (26.0%) | 9,339 | (24.8%) |  |
|  | | 2 | 17,262 | (29.2%) | 10,350 | (27.5%) |  |
|  | | 3 | 9,615 | (16.3%) | 5,690 | (15.1%) |  |
|  | | 4 | 9,890 | (16.8%) | 6,711 | (17.8%) |  |
|  | | 5 | 1,342 | (2.3%) | 1,246 | (3.3%) |  |
|  | | 6 | 3,001 | (5.1%) | 2,300 | (6.1%) |  |
|  | | 7 (more rural) | 2,596 | (4.4%) | 1,968 | (5.2%) |  |
| Previous TCM users | | | 12,395 | (18.8%) | 3,293 | (7.9%) | <0.001 |
| Co-morbidities | | |  |  |  |  |  |
|  | Hypertension | | 37,534 | (57.0%) | 26,528 | (64.0%) | <0.001 |
|  | Hyperlipidemia | | 22,777 | (34.6%) | 12,710 | (30.6%) | <0.001 |
|  | Heart failure | | 2,911 | (4.4%) | 2,754 | (6.6%) | <0.001 |
|  | IHD | | 12,086 | (18.4%) | 8,273 | (19.9%) | <0.001 |
|  | CVD | | 4,225 | (6.4%) | 4,311 | (10.4%) | <0.001 |
|  | Hyperuricemia | | 7,084 | (10.8%) | 4,930 | (11.9%) | <0.001 |
|  | COPD | | 7,971 | (12.1%) | 5,047 | (12.2%) | 0.79 |
|  | Charlson’s mortality index | | 3.6 | (2.0) | 4.1 | (2.0) | <0.001 |
|  | Modified DCSI score | | 1.3 | (1.2) | 1.5 | (1.3) | <0.001 |
| Confounding drugs | | |  |  |  |  |  |
|  | Diabetic drugs | |  |  |  |  |  |
|  | Insulin analogs | | 5,679 | (8.6%) | 4,577 | (11.0%) | <0.001 |
|  | Biguanides | | 35,258 | (53.6%) | 23,037 | (55.5%) | <0.001 |
|  | SU | | 40,961 | (62.2%) | 26,573 | (64.1%) | <0.001 |
|  | Alpha-glucosidase inhibitors | | 6,515 | (9.9%) | 4,329 | (10.4%) | 0.005 |
|  | TZD | | 9,039 | (13.7%) | 5,374 | (13.0%) | <0.001 |
|  | Others | | 4,297 | (6.5%) | 3,193 | (7.7%) | <0.001 |
|  | Lipid-lowering agent | |  |  |  |  |  |
|  | Statin | | 15,302 | (23.3%) | 9,443 | (22.8%) | 0.065 |
|  | Fibrate | | 7,663 | (11.6%) | 4,509 | (10.9%) | <0.001 |
|  | Others | | 178 | (0.3%) | 103 | (0.2%) | 0.49 |
|  | Anti-hypertensives | |  |  |  |  |  |
|  | ACEi | | 15,648 | (23.8%) | 11,043 | (26.6%) | <0.001 |
|  | ARB | | 15,012 | (22.8%) | 11,249 | (27.1%) | <0.001 |
|  | α-blocker | | 3,849 | (5.8%) | 3,136 | (7.6%) | <0.001 |
|  | β-blocker | | 18,486 | (28.1%) | 12,296 | (29.6%) | <0.001 |
|  | CCB | | 25,711 | (39.1%) | 18,843 | (45.4%) | <0.001 |
|  | Diuretics | | 15,235 | (23.1%) | 12,608 | (30.4%) | <0.001 |
|  | Vasodilator | | 7,883 | (12.0%) | 5,444 | (13.1%) | <0.001 |
|  | Central-acting agent | | 3,849 | (5.8%) | 3,136 | (7.6%) | <0.001 |
|  | Analgesics, aspirin | |  |  |  |  |  |
|  | NSAIDs | | 24,082 | (36.6%) | 12,420 | (29.9%) | <0.001 |
|  | COX-2 inhibitors | | 3,025 | (4.6%) | 2,055 | (5.0%) | 0.007 |
|  | Acetaminophen | | 20,117 | (30.6%) | 10,467 | (25.2%) | <0.001 |
|  | Aspirin | | 20,444 | (31.1%) | 14,281 | (34.4%) | <0.001 |
| Abbreviations: ACEi, angiotensin converting enzyme inhibitor; ARB, angiotensin II receptor blocker; CCB, calcium channel blocker; CCI, Charlson’s comorbidity index; COPD, chronic obstructive pulmonary disease; COX-2, cyclooxygenase-2 inhibitor; DCSI, Diabetes Complications Severity Index; NSAID, nonsteroidal anti-inflammatory drug; NTD, new Taiwan dollar; SU, Sulfonylureas; TCM, traditional Chinese medicine; TZD, Thiazolidinediones | | | | | | | |

**Table S5**. Comparable demographic features among TCM users and non-TCM users after 1:1 propensity score matching, in which caliper was set to 0.00001. The standard mean differences between two groups become smaller than the cohort matched by PSM with caliper 0.2 under this condition.

|  | | | TCM users  (n= 24,807) | | TCM nonusers  (n= 24,807) | | | Standardized mean difference |
| --- | --- | --- | --- | --- | --- | --- | --- | --- |
| Gender | | |  |  |  |  | | -0.009 |
|  | | Female | 11,547 | (46.5%) | 11,433 | (46.1%) | |  |
|  | | Male | 13,260 | (53.5%) | 13,374 | (53.9%) | |  |
| Age (years) | | |  |  |  |  | | 0.016 |
|  | | -20 | 27 | (0.1%) | 33 | (0.1%) | |  |
|  | | 21-40 | 811 | (3.3%) | 885 | (3.6%) | |  |
|  | | 41-60 | 8,032 | (32.4%) | 8,098 | (32.6%) | |  |
|  | | 61- | 15,937 | (64.2%) | 15,791 | (63.7%) | |  |
| Insured level (NTD/month) | | |  |  |  |  | | -0.003 |
|  | | 0-20,000 | 20,359 | (82.1%) | 20,348 | (82.0%) | |  |
|  | | 20,001-40,000 | 2,680 | (10.8%) | 2,664 | (10.7%) | |  |
|  | | 40,001- | 1,768 | (7.1%) | 1,795 | (7.2%) | |  |
| Geolocation | | |  |  |  |  | | 0.011 |
|  | | 1 (more urban) | 6,239 | (25.2%) | 6,275 | (25.3%) | |  |
|  | | 2 | 7,023 | (28.3%) | 7,108 | (28.7%) | |  |
|  | | 3 | 3,875 | (15.6%) | 3,891 | (15.7%) | |  |
|  | | 4 | 4,365 | (17.6%) | 4,284 | (17.3%) | |  |
|  | | 5 | 661 | (2.7%) | 669 | (2.7%) | |  |
|  | | 6 | 1,420 | (5.7%) | 1,395 | (5.6%) | |  |
|  | | 7 (more rural) | 1,224 | (4.9%) | 1,185 | | (4.8%) |  |
| Previous TCM users | | | 2,311 | (9.3%) | 2,238 | | (9.0%) | 0.009 |
| Co-morbidities | | |  |  |  | |  |  |
|  | Hypertension | | 14,998 | (60.5%) | 14,763 | | (59.5%) | 0.019 |
|  | Hyperlipidemia | | 8,176 | (33.0%) | 7,918 | | (31.9%) | 0.022 |
|  | Heart failure | | 1,329 | (5.4%) | 1,272 | | (5.1%) | 0.010 |
|  | IHD | | 4,844 | (19.5%) | 4,697 | | (18.9%) | 0.015 |
|  | CVD | | 1,853 | (7.5%) | 1,814 | | (7.3%) | 0.006 |
|  | Hyperuricemia | | 2,809 | (11.3%) | 2,821 | | (11.4%) | -0.002 |
|  | COPD | | 2,937 | (11.8%) | 2,890 | | (11.6%) | 0.006 |
|  | CCI | | 3.9 | (1.9) | 3.8 | | (1.9) | 0.022 |
|  | Modified DCSI score | | 1.4 | (1.2) | 1.3 | | (1.2) | 0.024 |
| Confounding drugs | | |  |  |  | |  |  |
|  | Diabetic drugs | |  |  |  | |  |  |
|  | Insulin analogs | | 2,384 | (9.6%) | 2,310 | | (9.3%) | 0.010 |
|  | Biguanides | | 13,502 | (54.4%) | 13,448 | | (54.2%) | 0.004 |
|  | SU | | 15,619 | (63.0%) | 15,589 | | (62.8%) | 0.003 |
|  | Alpha-glucosidase inhibitors | | 2,474 | (10.0%) | 2,447 | | (9.9%) | 0.004 |
|  | TZD | | 3,283 | (13.2%) | 3,276 | | (13.2%) | 0.001 |
|  | Others | | 1,761 | (7.1%) | 1,717 | | (6.9%) | 0.007 |
|  | Lipid-lowering agent | |  |  |  | |  |  |
|  | Statin | | 5,812 | (23.4%) | 5,563 | | (22.4%) | 0.024 |
|  | Fibrate | | 2,893 | (11.7%) | 2,769 | | (11.2%) | 0.016 |
|  | Others | | 59 | (0.2%) | 66 | | (0.3%) | -0.005 |
|  | Anti-hypertensives | |  |  |  | |  |  |
|  | ACEi | | 6,313 | (25.4%) | 6,170 | | (24.9%) | 0.013 |
|  | ARB | | 6,207 | (25.0%) | 6,034 | | (24.3%) | 0.016 |
|  | α-blocker | | 1,626 | (6.6%) | 1,600 | | (6.4%) | 0.004 |
|  | β-blocker | | 7,344 | (29.6%) | 7,021 | | (28.3%) | 0.029 |
|  | CCB | | 10,509 | (42.4%) | 10,232 | | (41.2%) | 0.023 |
|  | Diuretics | | 6,618 | (26.7%) | 6,413 | | (25.9%) | 0.019 |
|  | Vasodilator | | 3,118 | (12.6%) | 3,025 | | (12.2%) | 0.011 |
|  | Central-acting agent | | 2,426 | (9.8%) | 2,404 | | (9.7%) | 0.003 |
|  | Analgesics | |  |  |  | |  |  |
|  | NSAID | | 8,036 | (32.4%) | 7,908 | | (31.9%) | 0.011 |
|  | COX-2 inhibitors | | 1,224 | (4.9%) | 1,193 | | (4.8%) | 0.006 |
|  | Acetaminophen | | 6,788 | (27.4%) | 6,601 | | (26.6%) | 0.017 |
|  | Aspirin | | 8,154 | (32.9%) | 7,854 | | (31.7%) | 0.026 |
| Abbreviations: ACEi, angiotensin converting enzyme inhibitor; ARB, angiotensin II receptor blocker; CCB, calcium channel blocker; CCI, Charlson’s comorbidity index; COPD, chronic obstructive pulmonary disease; COX-2, cyclooxygenase-2 inhibitor; DCSI, Diabetes Complications Severity Index; NSAID, nonsteroidal anti-inflammatory drug; NTD, new Taiwan dollar; SU, Sulfonylureas; TCM, traditional Chinese medicine; TZD, Thiazolidinediones | | | | | | | | |

**Figure S1.** Competing analysis on ESRD in the matched groups, by TCM users and nonusers. Caliper was set to 0.00001 when performing PSM and the result was similar to the cohort matched by using PSM with caliper 0.02.


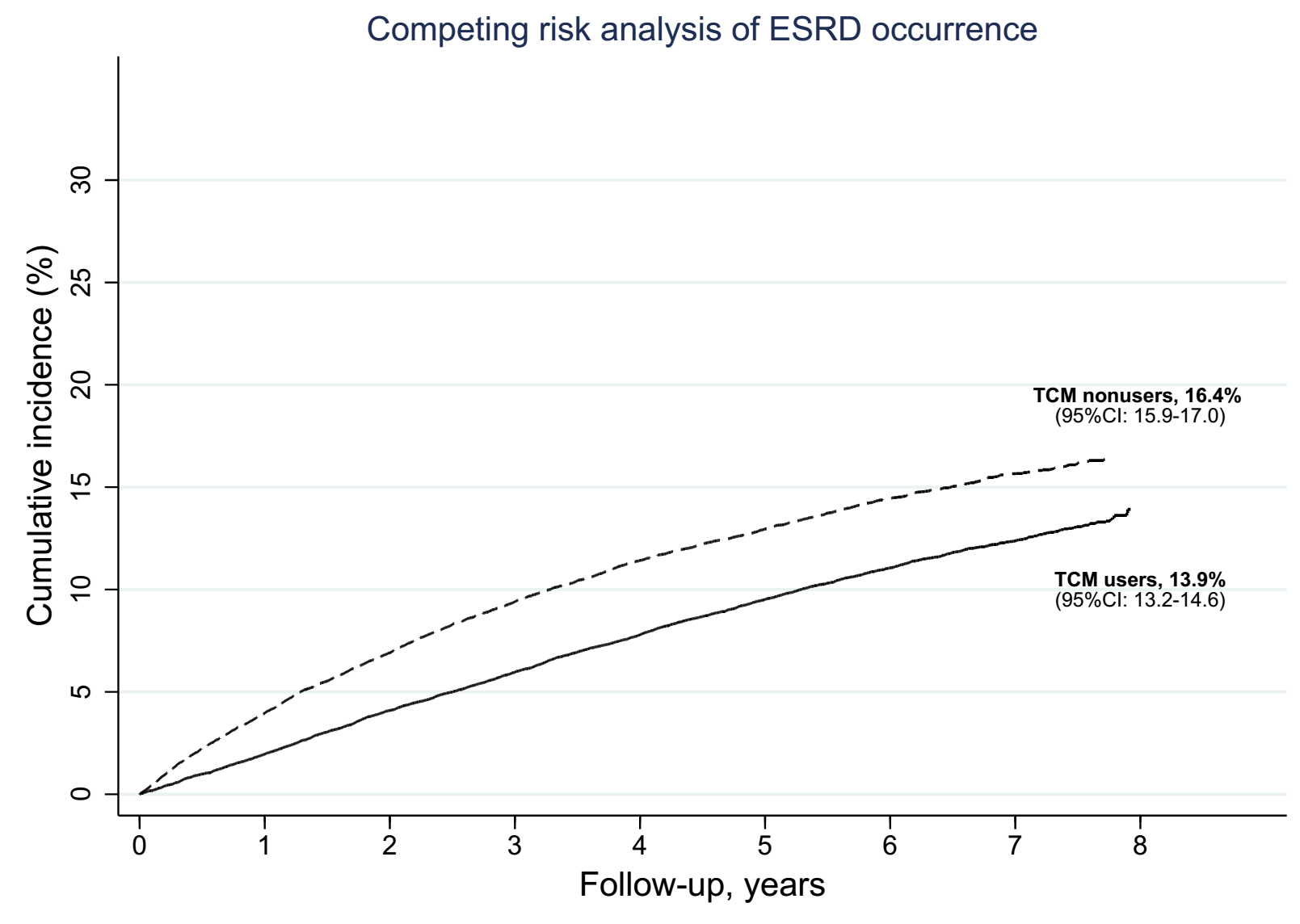


**Figure S2.** Survival analysis on mortality rate in the matched groups, by TCM users and nonusers. Caliper was set to 0.00001 when performing PSM and the result was similar to the cohort matched by using PSM with caliper 0.02.


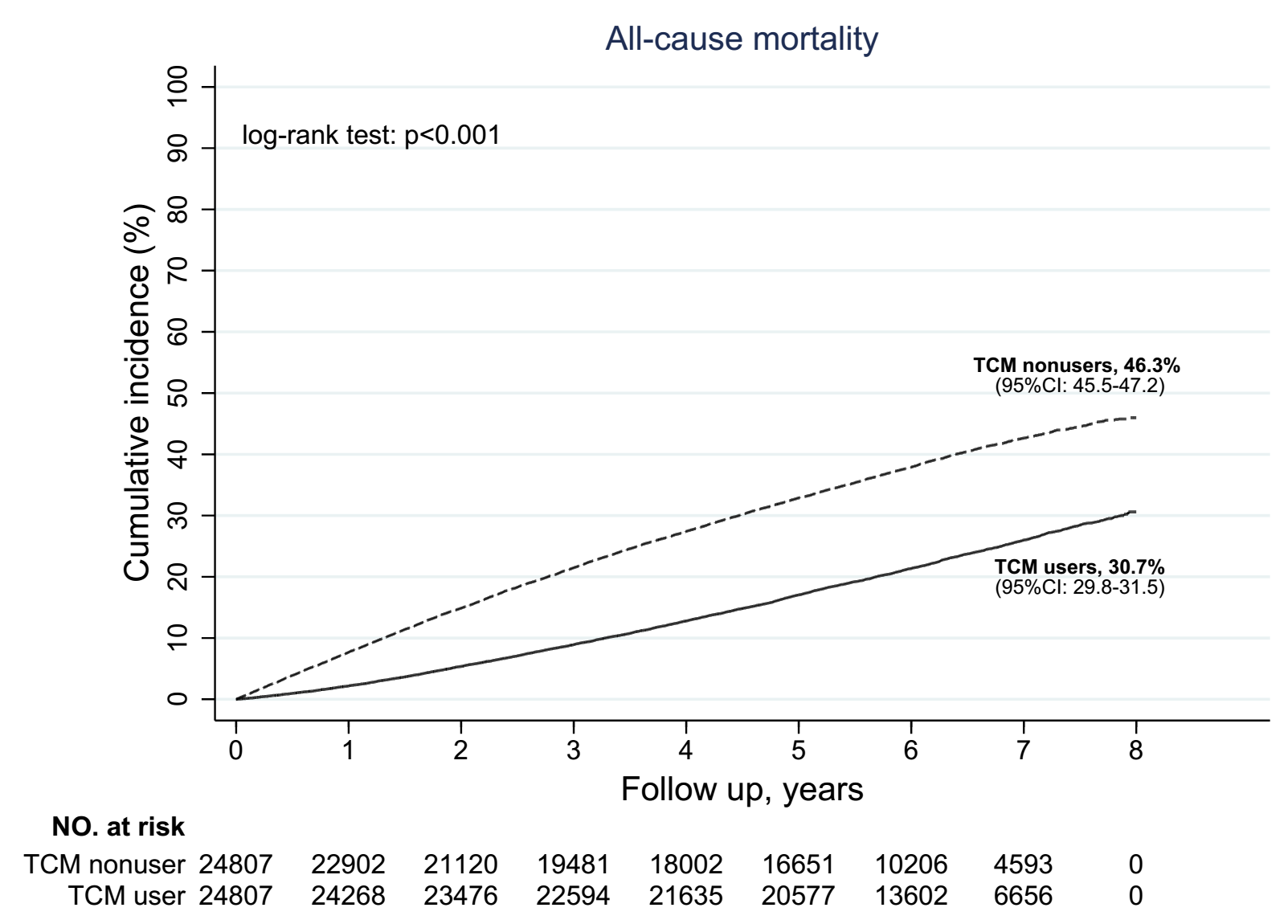

Supplement: Supplementary file 1 — Table S1. Diagnosis codes used in the study. Table S2. Medications codes used in this study. Table S3. The CKD stage and albuminuria of DN patients when diagnosed in the Department of Nephrology, Chang Gung Memorial Hospital, Taiwan, from 2004 to 2012. (n = 5384 with 136 normoalbuminuria patients). Table S4. Demographic and medical history of patients with incident diabetic nephropathy during 2004–2006 (N = 107,294). Table S5. Comparable demographic features among TCM users and non-TCM users after 1:1 propensity score matching, in which caliper was set to 0.00001. The standard mean differences between two groups become smaller than the cohort matched by PSM with caliper 0.2 under this condition. Figure S1. Competing analysis on ESRD in the matched groups, by TCM users and nonusers. Figure S2. Survival analysis on mortality rate in the matched groups, by TCM users and nonusers. (DOCX 335 kb) [file 12906_2019_2491_MOESM1_ESM.docx]
